# Profiling Chemobiological Connection between Natural Product and Target Space Based on Systematic Analysis

**DOI:** 10.3390/ijms241411265

**Published:** 2023-07-10

**Authors:** Disheng Wang, Xue Li, Yicheng Miao, Qiang Zhang

**Affiliations:** Shaanxi Key Laboratory of Natural Products & Chemical Biology, College of Chemistry & Pharmacy, Northwest A&F University, Yangling 712100, China

**Keywords:** biogenetic pathway, chemical space, target feature, data analysis

## Abstract

Natural products provide valuable starting points for new drugs with unique chemical structures. Here, we retrieve and join the LOTUS natural product database and ChEMBL interaction database to explore the relations and rhythm between chemical features of natural products and biotarget spaces. Our analysis revealed relations between the biogenic pathways of natural products and species taxonomy. Nitrogen-containing natural products were more likely to achieve high activity and have a higher potential to become candidate compounds. An apparent trend existed in the target space of natural products originating from different biological sources. Highly active alkaloids were more related to targets of neurodegenerative or neural diseases. Oligopeptides and polyketides were mainly associated with protein phosphorylation and HDAC receptors. Fatty acids readily intervened in various physiological processes involving prostanoids and leukotrienes. We also used FusionDTA, a deep learning model, to predict the affinity between all LOTUS natural products and 622 therapeutic drug targets, exploring the potential target space for natural products. Our data exploration provided a global perspective on the gaps in the chemobiological space of natural compounds through systematic analysis and prediction of their target space, which can be used for new drug design or natural drug repurposing.

## 1. Introduction

Studies have shown that natural products possess enhanced binding specificity, lower preclinical toxicity, and more favorable advancement during clinical trials [1]. These features may be attributed to the rich stereochemistry of natural product structures. Furthermore, exploring molecules with “beyond-rule-of-5” properties, common in natural products, has been considered a potential avenue for drug discovery. Therefore, natural products provide potential opportunities for modern drug discovery, including improving binding specificity and clinical trial progression [2].

Defining and exploring chemical spaces is essential for facilitating the process of molecular discovery and can be operationalized through algorithmic approaches and machine learning. Chemical space refers to the complete collection of all conceivable molecules or materials, with subspaces that are more restricted in scope because of the structures or functions of the molecules they comprise [3]. The chemical space of natural products contains many different chemotypes, including lipids, alkaloids, sugars, flavonoids, polyketides, oxosteroids, coumarins, psoralenes, steroidal or cardiac glycosides and oligopeptides [1].

The importance of chemical space lies not only in its size but also in its diversity. This is especially true when the space contains molecules that meet the design objectives for various discovery tasks [4]. To achieve a diverse chemical space, it is important to select building blocks with unique or intriguing structural motifs. Simply relying on typical chemical processes may not produce enough compounds to adequately test. By prioritizing diversity, scientists tend to increase the likelihood of discovering effective molecules that can fulfill various discovery objectives.

Pharmaceuticals contain chemical and biological aspects. Biological target space refers to all possible biological targets a drug could interact with [5]. There is a close relationship between chemical and biological target spaces, as chemical structures must be designed and optimized to interact with specific biological targets to achieve desired therapeutic effects. In drug discovery, scientists have created various computational methods to effectively and logically explore chemical space such as molecular generation [6] and chemical similarity metrics [7]. Similarly, target-based and virtual screening methods can help identify potential biological targets for a given chemical compound or library, such as a natural product library. Thus, integrating and exploring chemical and target spaces through computational methods can expedite the discovery of promising drug candidates.

The chemobiological connection between the chemical space of natural products and their target space combines information about the interactions between bioactive compounds and human proteins. Analyzing and predicting these interactions can help design new drugs and nutraceuticals. There is a significant lack of research in the chemobiological space of natural products, resulting in numerous gaps in knowledge about their chemical and biological properties.

To explore the relationship between chemistry and biology, cheminformatic techniques and target prediction methods can be employed to identify potential interactions for a large proportion of bioactive compounds. The analysis of target space is essential to understand the biological effects of natural products at a molecular level and identify new opportunities for drug discovery. In addition, a deep learning model was utilized to predict the affinity between ligands and proteins, resulting in a larger number of potential interactions between natural products and biotargets of approved drugs.

**Figure 1:**
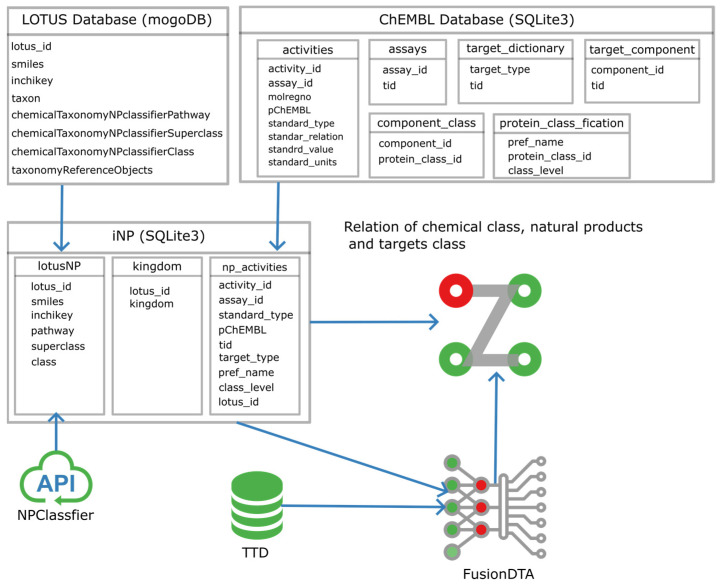
Scheme to profile chemobiological connections among natural sources, natural products and biotargets.

Herein, we profiled the chembiological connections among bioresource taxons, natural products and biotargets by combining and analyzing the database of ChEMBL [8] and LOTUS natural product library [9]. The potential target space was also predicted by the deep learning model FusionDTA [10] to profile the link between natural product space and therapeutic targets’ clinical usages (TTD targets) [11]. The overall data analysis pipeline was gathered, as shown in Figure 1.

## 2. Results

### 2.1. LOTUS Natural Products Analysis

**Figure 2:**
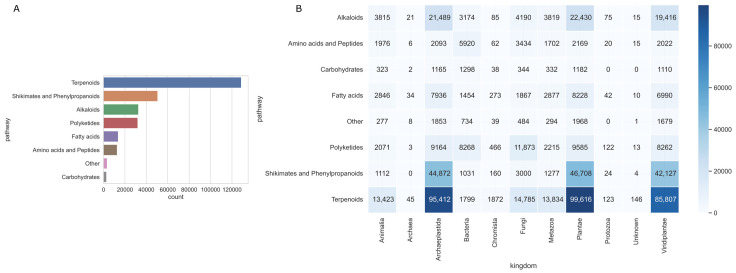
Count of LOTUS natural products library. (**A**) Number of natural products in different pathways. (**B**) Number of natural products of different pathways and the kingdom of plants.

We fixed missing information on LOTUS natural product classifications by connecting to NPClassfier [12]. However, there were still 3405 compounds for which NPclassfiecould not determine structural type information, so we classified them as “other” type. As shown in Figure 2A, the number of terpenoids dominates at 128,700, followed by 50,613 compounds in the shikimates acid and phenylpropanoids acid pathway, and 32,634 and 31,971 compounds in the alkaloids and polyketides categories, respectively. As shown in Figure 2B, terpenoids, phenylpropanoids and alkaloids mainly distribute in the taxon kingdoms of Archaeplastida, Plantae and Viridiplantae. Polyketides originated mainly from Fungi, Archaeplastida, Plantae, Viridiplantae and Bacteria kingdoms. Peptides were mainly from Bacteria. Archaeplastida, Plantae and Viridiplantae were three kingdoms of the most chemically diverse and major sources of natural compounds.

### 2.2. Activity Values Distribution of Natural Product

**Figure 3:**
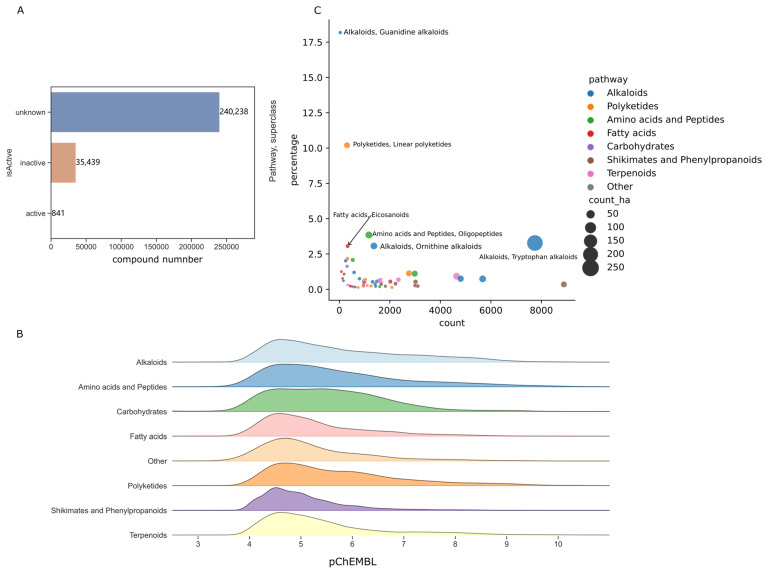
(**A**) Active and inactive natural products based on combining analysis of LOTUS and ChEMBL database, including all target types (single protein, protein complex, cell lines, tissue, etc.). (**B**) Distribution of pChEMBL values of natural products. (**C**) Percentage of high active sup class of natural product (pChEMBL > 8, only activity data on single proteins). Complete data associated can be found in datasheet S1 in Supplementary Materials.

The ChEMBL database does not classify its recorded compounds into categories of natural and non-natural products. The activity records of the natural products (354,795 entries) that were selected are here associated with the LOTUS database, and the activity intensities in ChEMBL were mathematically processed to pChEMBL. Concentration-response activity values, such as IC_50_, EC_50_, XC_50_, AC_50_, K_i_, K_d_, and potency, are expressed as a negative logarithm known as pChEMBL. In constructing many machine learning models for predicting activity [13], pChEMBL is often used to classify compounds as active or inactive. Based on the available activity records in the ChEMBL and the chemical items in the LOTUS database, the number of natural products with documented high activity (pChEMBL > 8) in the ChEMBL database was 841 (Figure 3A), including activity results tested on animals, tissues, cells, mixed enzymes and single proteins. Another 35,439 compounds had activity assay result data, but no high activity was found. The remaining majority of 240,238 (13.1%) natural products had no activity records in the ChEMBL database.

We then used a joyplot to display the kernel density distribution of the activity intensity (pChEMBL) along the biogenic pathway of natural products (Figure 3B). We used kernel density estimation (KDE) in the plot to visualize the pChEMBL distribution. The KDE method is used to estimate the probability density function of random variables. It is widely utilized in machine learning, data analysis, and statistics to estimate the underlying probability distribution from data points. The resulting curve can provide a smooth estimate of the probability density function that can be used for further analysis or modeling.

From the pChEMBL KDE plots (Figure 3B), it is clear that most activity intensities for all the categories of natural products were concentrated between 4 and 6. Here, we use pChEMBL > 8 as the defining threshold for highly active substances, corresponding to an IC_50_ or EC_50_ less than 10 nM. Compounds with this activity level can often be used as lead compounds or candidates for further drug development.

Usually, natural products are classified by the biogenic route. We used the NPClassfier to supplement the missing classification information in the LOTUS database. They were classified into three levels, pathway, superclass and class. Unclassifiable compounds are defined as type Other.

We then used the activity data with “single protein” and counted the number of highly active substances in each superclass of natural product (pChEMBL > 8). We counted the percentage of high activities of natural products based on the total number in each pathway category. As shown in Figure 3C, guanidine and linear polyketide had significantly higher hit rates (18.18% and 10.20%) than the other categories. Natural products originating from the pathways of amino acids, fatty acids and phenylpropanoids had a higher hit rate of more than 1.0%.

### 2.3. Target Space of Natural Products of High Hit Rate Superclass

**Figure 4:**
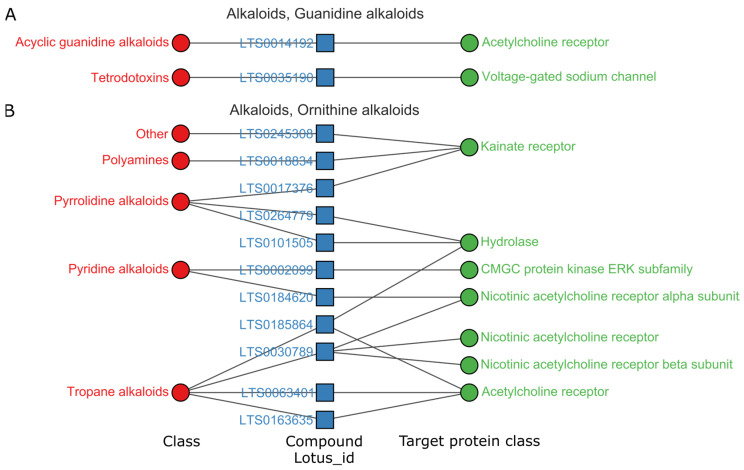
Relations among targets, compounds and natural product class of guanidine (**A**) and ornithine (**B**), pChEMBL > 8, target protein class levels (≥2). Full data associated can be found in datasheet S2 in Supplementary Materials.

**Figure 5:**
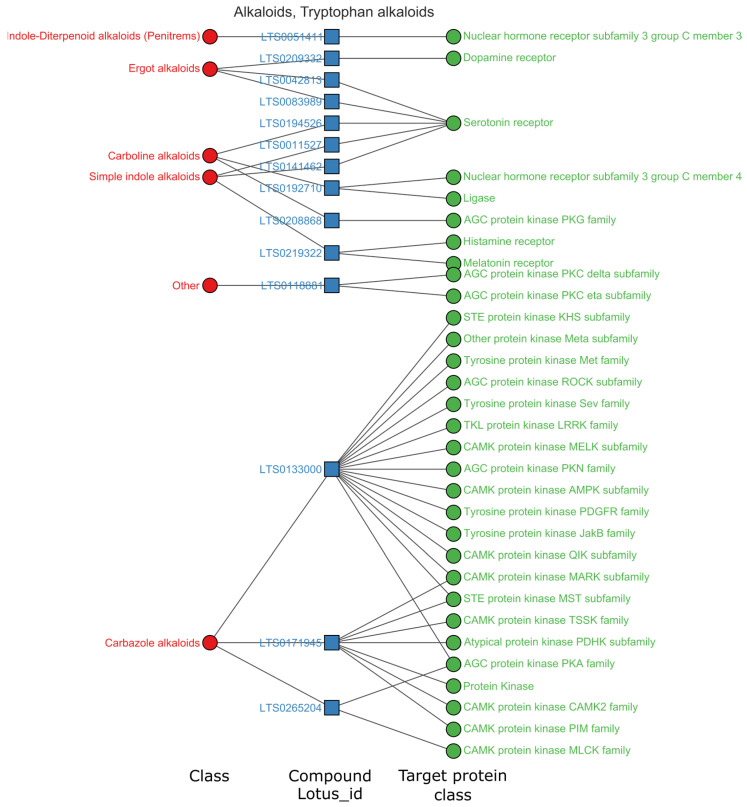
Relations among targets, compounds and chemical structure class of tryptophan alkaloids, pChEMBL > 9, target protein class levels (≥2). Complete data associated can be found in datasheet S2 in Supplementary Materials.

**Figure 6:**
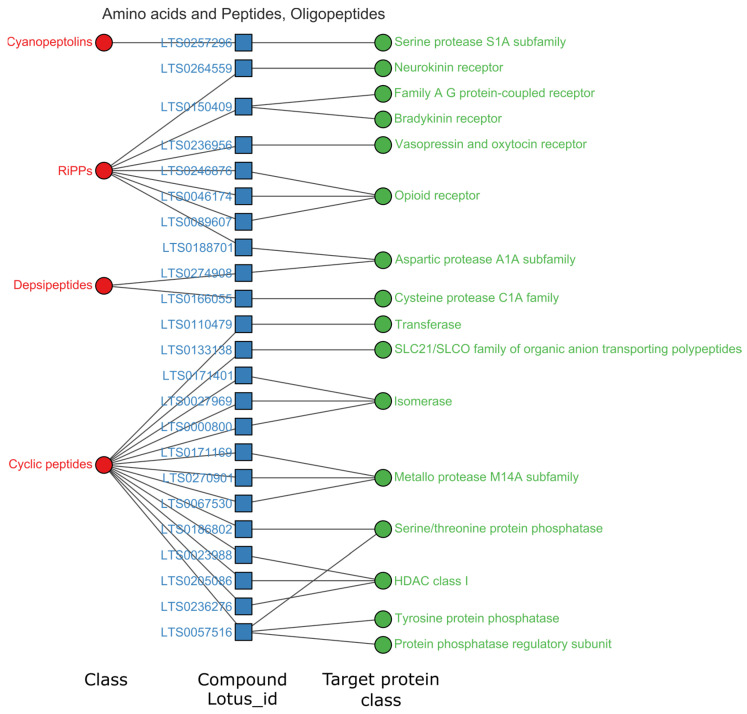
Relations among targets, compounds and chemical structure class of Oligopeptides, pChEMBL > 8. Full data associated can be found in datasheet S2 in Supplementary Materials.

**Figure 7:**
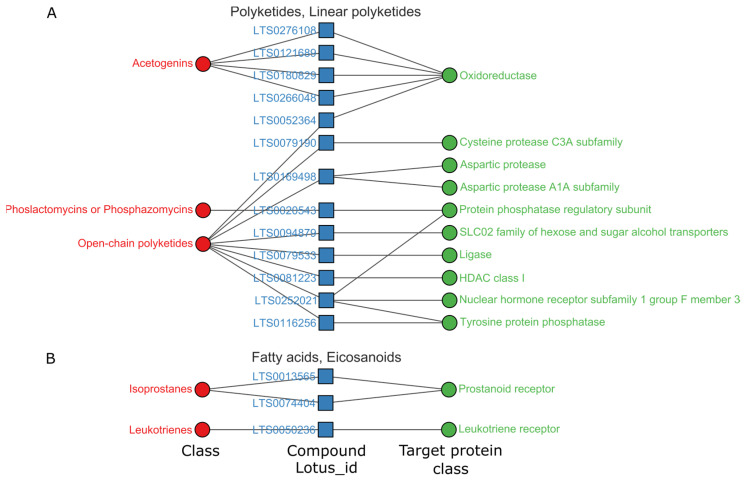
Relations among targets, compounds and chemical structure classes of linear polyketides (**A**) and eicosanoids (**B**), pChEMBL > 8. Complete data associated can be found in datasheet S2 in Supplementary Materials.

We selected the superclasses with the highest hits for further data analysis (hit rate > 3%). In total, 825 high-activity items (pChEMBL > 8) were acquired on single proteins. As shown in Figure 3C, the high hit rate superclasses were guanidine, linear polyketide, oligopeptide, tryptophan, ornithine and eicosanoid. Patterns between the natural chemical class, natural compounds and their target classes were visualized in network plots, as shown in Figure 4, Figure 5, Figure 6 and Figure 7.

**Figure 8:**
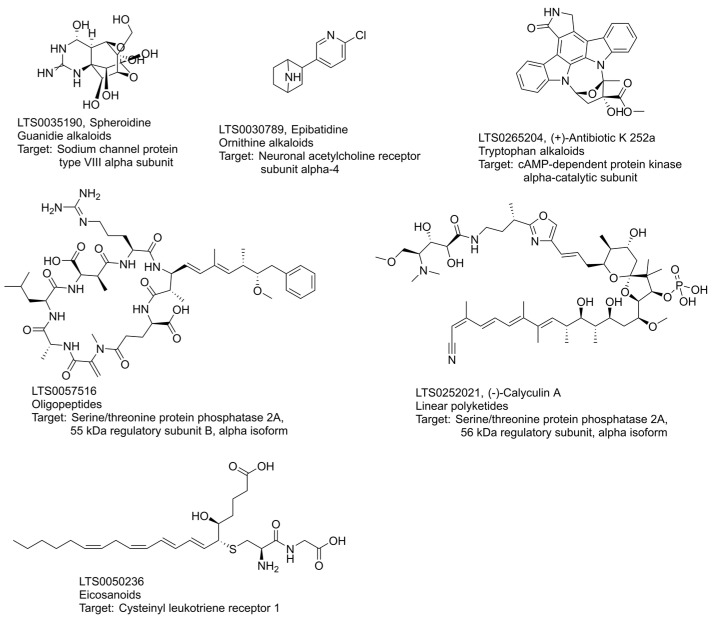
Chemical structures and targets of highest active molecules in high hit rate superclasses. Data were retrieved from ChEMBL and LOTUS databases.

There were 415 activity entities of alkaloids with high activities on a single protein (sheet S2 in Supplementary Materials). Guanidine, ornithine and tryptophan alkaloids gave a high hit rate of more than 3.0%. Figure 4 demonstrates the connection among the classes of guanidine and ornithine, chemical compounds and targets. Guanidines and ornithines acted mainly on receptors in the central nervous system, such as acetylcholine receptors and sodium channels. Trtradotoxin mainly acted on sodium channel proteins, such as LTS0035190 (Figure 8). LTS0035190 has a potential interaction with the α subunit of sodium channel protein type VIII [14]. Tropine acted exclusively on the muscarinic or neuronal acetylcholine receptor family, such as the compound LTS0030789 (Figure 8), targeting neuronal acetylcholine receptor α4. Trtradotoxin and Tropane were the typical natural leads for developing highly selective neurological drugs.

Tryptophan alkaloids also acted on the nervous system with unique targeting features. According to the data of high activity related tryptophan alkaloids class (datasheet S2 in Supplementary Materials), there were 254 activity entries covering 14 natural chemical classes, 45 compounds, and 172 targets. The biotargets belong to 87 protein classes. As shown in Figure 5, the primary targets were the serotonin receptor (41 entries), adrenergic receptor (21 entries), and dopamine receptor (10 entries). It was obvious that serotonin and adrenergic receptors were the two main target classes for tryptophan alkaloids. The natural ligands for serotonin receptors were primarily ergot alkaloids (24 entries, 7 compounds) and simple indole alkaloids (14 entries, 4 compounds). The compound class targeting adrenergic receptors were mainly ergot alkaloids (14 entries), yohimbine-like alkaloids (5 entries), and phenylalanine-derived alkaloids (2 entries). Network diagrams can show correlations between different data sets but are limited in the data items they can present. To present the most strongly active pattern, we selected entries with pChEMBL > 9. Figure 5 shows 14 tryptophan alkaloids in indole diterpenoid, ergot, carboline, simple indole and carbazole classes. The compound LTS0133000 and LTS0171945 had more targets than other alkaloids. LTS0133000 had been recorded with 15 target families (pChEMBL > 9), mainly acting on tyrosine protein kinase families and other protein kinases. LTS0171945 (staurosporine) had 7 target families, primarily acting on CAMK protein kinases. In superclass tryptophan, compound LTS0265204 has the highest affinity to the α-catalytic subunit of cAMP-dependent protein kinase according to ChEMBL records.

The natural products of oligopeptides had 45 high-activity entries. As shown in Figure 6, oligopeptides mainly targeted isomerase (three compounds, six entries), opioid receptors (three compounds, six entries), and HDAC class I (three compounds, five entries). These active molecules of the oligopeptides are concentrated in cyclic peptides, RiPPs, depsipeptides and cyanopeptides. Cyclic peptides mainly targeted isomerase, HDAC class I, metalloprotease, protein phosphatase, and serine/threonine protein phosphatase. Peptides of RiPPs primarily showed high activities on the targets of the opioid receptor, bradykinin receptor and vasopressin and oxytocin receptor. Depsipeptides showed high activities on targets of aspartic protease and cysteine protease. In oligopeptides, the compound LTS0057516 showed higher affinity to the 55 kDa regulatory subunit B α isoform of serine/threonine protein phosphatase 2A [15]. The compound also had multi-targets of protein phosphatase, tyrosine protein phosphatase, and serine/threonine protein phosphatase [15,16,17].

The linear polyketides superclass had 31 records of high activity and contained 22 chemical structures, which included 15 open-chain polyketides, 5 acetogenins, 1 phosphazomycin and 1 linear polyene, as shown in Figure 7A. Open-chain polyketides acted primarily on protein phosphatases, such as tyrosine protein phosphatase and proteins of HDAC class I. Acetogenins mainly acted on oxidoreductase. Phosphazomycin acted on protein phosphatases. In the superclass of linear polyketides, the compound LTS0252021 acted on target classes of protein phosphatase and nuclear hormone receptors [15,17]. The compound LTS0252021 shows high activity on the 56 kDa regulatory subunit B α isoform of serine/threonine protein phosphatase 2A [17].

Eicosanoids are another class of highly active linear molecules, with 10 records of high activity and 4 compounds. We restricted the classification of target protein classes to two or more. The network diagram (Figure 7B) showed three of these compounds. These three compounds belong to the isoprostanes and leukotrienes class of fatty acids, respectively, targeting prostanoid and leukotriene receptors. In the eicosanoid superclass, the compound LTS0050236 has high activity on cysteinyl leukotriene receptor 1 [18,19].

### 2.4. Large-Scale Virtual Screening Based on the Deep Learning Model FusionDTA

The activity of most natural products is not recorded (Figure 3A). We assessed the potential target space by predicting the affinity between small molecules and protein targets to profile the target space for all-natural products in the LOTUS database. We downloaded 622 target sequences from TTD that have been successfully used in clinical therapeutics and have the most application value. However, it is too computationally expensive for traditional molecular docking methods to calculate all pairwise affinities between the large-scale small molecule dataset and many protein targets. Furthermore, each protein molecule must be pre-processed manually for binding pockets and cleaning excess side chains. Therefore the use of traditional molecular docking methods cannot accomplish our task.

Recently, there has been a rapid emergence of deep learning-based models for predicting affinity. These methods are extremely fast on the GPU and rely solely on the SMILES strings of small molecules and protein sequences to obtain predicted affinity values more reliably. These deep learning models have not needed pre-processing and optimization conformations for small or big molecules. Thus they are well suited to the computational task herein. After a comparative evaluation, we chose the FusionDTA [10] model optimized by knowledge distillation for further data exploration. The performance of the drug-target interaction prediction model is improved by FusionDTA’s innovative multi-head linear attention mechanism. The mechanism aggregates global information using attention weights, rather than relying on max-pooling to select the largest one, resulting in better outcomes [10].

**Figure 9:**
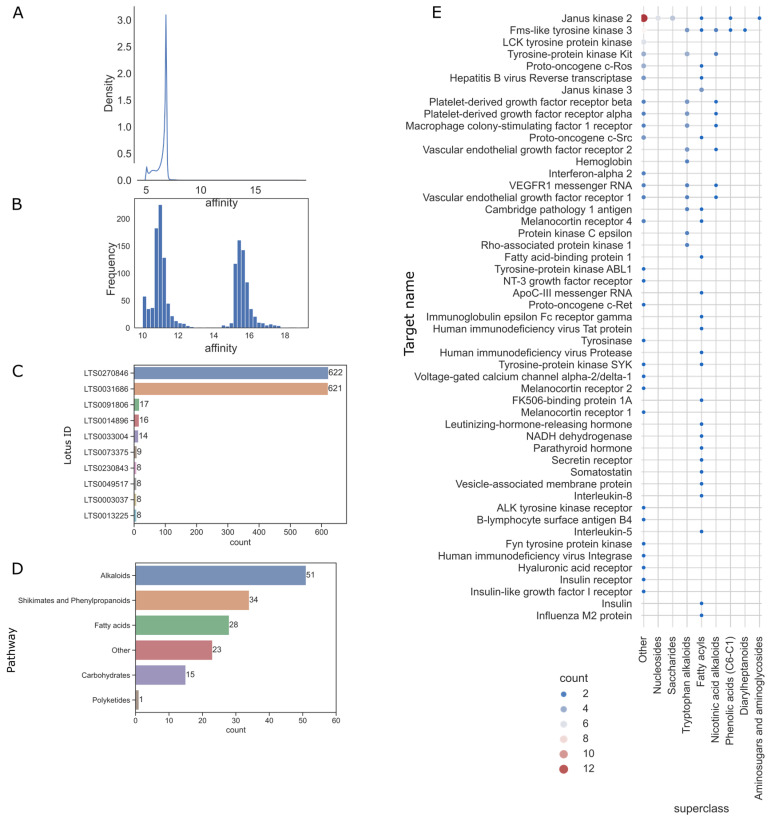
Deep learning model (FusionDTA) based affinity prediction for natural products and therapeutical targets in clinical usage. (**A**) Data distribution of predicted affinity. (**B**) Top counts of potential targets of natural products with an affinity of more than 10. (**C**) Count of potential targets in natural product pathway. (**D**) Potential targets superclass of natural products and target. Complete data associated can be found in datasheet S3 in Supplementary Materials.

The targets set contains 622 therapeutic targets in clinical application. The predictions showed that the affinities were mainly concentrated around 6.8, as shown in Figure 9A. The high-affinity values distribution was gathered in Figure 9B. These affinity values were concentrated in the two intervals of 10–12 and 15–17. We thus selected items with an affinity of more than 10.0 as effective interactions. Two molecules (LTS0270846 and LTS0031686 in Figure 9C) showed affinities to almost all proteins. These two compounds are sulfur and hexathiepane, probably due to the high content of S elements in them. We excluded these two abnormal and non-selective compounds and, thus, obtained 152 molecules with high affinity from this virtual screen. All 152 natural molecules were counted by categories of pathways, as shown in Figure 9D. Alkaloids had the highest number (51) of high-affinity items. We then summarised the number of correspondences between the superclass and the target (Figure 9E), the tryptophan alkaloids and fatty acyls have the broadest affinity spectrum.

## 3. Discussion

### 3.1. Biogenic Pathways of Natural Products and Bioresources Species Tendencies

Usually, chemical structures are classified based on the functional groups and structure scaffolds. Deep learning models have also been trained in recent years, such as ClassyFire [20], which can automatically classify chemical structures at multiple levels. However, unlike simple synthetic compounds, natural products have many functional groups and an unusually variable skeletal structure. Despite their structural complexity and variability, natural products have limited biogenic pathways and biogenic reactions in their biosynthesis. Therefore, the chemical classification from a biogenic perspective is well suited to the structural characteristics of natural products. Our statistics showed a clear tendency between the classification of broad categories of biogenesis and source taxa. Bacteria and fungi, for example, were more likely to produce polyketides, while terpenoids were dominant in plants and Archaeplastida.

The close link between the bioresources and natural pathways could help us understand botanicals’ intrinsic functions in different taxa. According to our statistics (Figure 2B), plants were more chemically diverse than other species. They had a greater abundance of terpenoids, phenylpropanoids, and Alkaloids. So, they had the potential for a broader range of biological activities. From a data point of view, which explains why herbal medicines are primarily of plant rather than animal origin.

### 3.2. Chemical Diversity and High Activities Hit Rates

Terpenoids, phenylpropanoids, and alkaloids were the most prominent structural families of natural products, and they possessed not only a large number of chemicals but also high chemical diversities [21,22,23]. However, according to our statistics (Figure 3C), although terpenoids have the highest chemical diversity, the hit rate of each subfamily in terpenoids was not as prominent as that of alkaloids and polypeptide superclass. Nitrogenous compounds dominated all pathways with a hit rate of more than 3.0%, including alkaloids, peptides, and nitrogenous fatty acids (Figure 8). Chemical diversity is indeed a critical factor in drug development. However, the low hit rate of terpenoids suggested that not all diversity is so necessary. We summarised the most active compounds from each superclass with a high hit rate (>3%), as shown in Figure 8. All compounds, without exception, contained nitrogen atoms. It was clearly demonstrated that nitrogen is an essential factor for highly active natural products. Therefore, seeking nitrogen-containing natural products could be easier to find highly active molecules. Alternatively, functional repurposing for natural nitrogen-containing compounds might likely reveal better activity.

### 3.3. Privilege Targets of Natural Products in Superclass with High Hit Rates

We have observed that natural products sharing the same origins tend to have similar active targets. For instance, various subtypes of tryptamine alkaloids possess activity on CAMK kinases. Hence, when searching for new functions of natural products, we can use similar origin categories as a guide. The connection between biotargets and diseases is quite intricate; thus, we provided herein a concise summary of the categories of natural products with high hit rates, biotargets, relevant diseases and mechanisms.

#### 3.3.1. Alkaloids Mainly Regulate the Central Nervous System

Three superclasses of alkaloids had high activities with a hit rate of more than 3%, guanidine, tryptophan and ornithine. Their chemical scaffolds and targets differed, but they all acted on the nervous system. The mainly involved targets of alkaloids were voltage-gated sodium channels and receptors of acetylcholine, serotonin (5-HT), adrenergic, and dopamine.

Guanidine alkaloids acted on voltage-gated sodium channels with high affinities. There are various diseases called channelopathies that are associated with voltage-gated sodium channels. These diseases may be caused by genetic mutations or acquired dysfunction due to tissue injury or autoimmune diseases. Sodium channels are a primary target for therapeutic medications, including drugs used for local anesthesia, epilepsy, chronic pain, and cardiac arrhythmia [24].

Both guanidine and ornithine alkaloids acted on acetylcholine receptors. Tropane alkaloids acted on nicotinic acetylcholine receptors. Recently, the α7 nicotinic acetylcholine receptor was reported to be associated with a range of neural diseases, including Alzheimer’s disease, schizophrenia, anxiety, depression and inflammation [25].

Tryptophan alkaloids primarily acted on serotonin (5-HT), adrenergic, and dopamine receptors. Recent research suggests that the 5-HT7 receptor (5-HT7R) may be linked to neurodegenerative diseases such as Alzheimer’s, Parkinson’s, and Huntington’s disease. The potential role of 5-HT7R in neurodegenerative diseases is still not well understood. Studies conducted in animals have indicated that targeting 5-HT7R could serve as a potential therapy for these disorders, as it has been shown to have neuroprotective properties [26]. In addition, Aβ-mediated neurodegeneration, oxidative stress, apoptosis, and inflammation are biological mechanisms involved in neurodegeneration. Therefore, 5-HT7R may have a potential role in the pathophysiology of several neurodegenerative diseases [27]. Serotonin (5-HT) can also interact with gut microbiota and its implications for host physiology, relating to metabolic dysfunctions such as obesity, hepatic steatosis, and glucose intolerance [28].

Adrenergic receptors are implicated in several diseases, including Alzheimer’s disease. In Alzheimer’s disease, dysregulation of the noradrenergic system, particularly the degeneration of locus coeruleus noradrenergic neurons, has been linked to its development [29]. Several adrenergic receptors, including α or β adrenergic receptors, have been studied in Alzheimer’s disease mice models, where their blockade or activation has been shown to affect cognitive functions and the processing and secretion of Aβ and tau pathology [30].

Dopamine is involved in various neurological and neuropsychiatric diseases, including Parkinson’s disease, addiction and schizophrenia. These illnesses are linked to irregularities in dopamine neurotransmission and interruptions in brain circuits where dopamine is essential. [31]. Impulsive control disorders, such as hypersexuality, have also been associated with dopamine-related diseases like Parkinson’s disease [32].

#### 3.3.2. Oligopeptides and Linear Polyketides Targeted HDAC Class I

Both oligopeptides and linear polyketides acted on HDAC (histone deacetylases) class I proteins. The HDAC class plays a crucial role in regulating epigenetics by controlling the acetylation of lysine side chains in histone tails. Studies show that HDACs exhibit abnormal function and expression in various tumor cells, like breast, lung, liver and gastric cells. HDACs also play a significant role in neurodegenerative diseases such as Alzheimer’s disease, Huntington’s disease, Parkinson’s disease, and mood disorders. Moreover, HDACs are also involved in HIV infection, kidney diseases, and inflammatory diseases [33]. Thus, HDAC inhibitors show great potential in treating these diseases by altering these abnormalities.

Oligopeptides can also act on opioid receptors. Opioids are a class of drugs traditionally used for pain management, affecting the µ, κ and δ opioid receptors in the central and peripheral nervous system. However, opioids may lead to adverse effects like tolerance, addiction, respiratory depression, constipation, nausea and drowsiness [34].

#### 3.3.3. Eicosanoids Acted Primarily on Prostanoid and Leukotriene Receptors

Prostanoid and leukotriene play crucial roles in numerous biological processes and diseases. The receptors for prostanoids are significant in regulating the immune system, managing allergic and inflammatory responses, mobilizing dendritic cells, and affecting PGD2-induced sleep [35]. They are also associated with developing skin papilloma and activating multiple signaling pathways [36]. Regarding diseases, prostanoid receptors have been linked to migraine, where lymphocytes in migraine patients were found to have heightened β-adrenoceptor and prostacyclin responsiveness. Research has indicated that prostaglandin signaling plays a role in inflammation-related illnesses like rheumatoid arthritis and inflammatory bowel disease [35].

Leukotriene receptors have been linked to various health conditions, including cerebral ischemia, Alzheimer’s disease, Parkinson’s disease, airway inflammation, cancer and cardiovascular disorders. These receptors play a crucial role in the body’s inflammatory response by facilitating leukocyte chemotaxis, vascular leakage, and astrocyte proliferation. There are two types of leukotriene receptors, CysLT1 and CysLT2, which operate through distinct signaling pathways. CysLT1 functions through phospholipase C and Ca^2+^ mobilization, while CysLT2 mediates the inflammatory response through Gq/Gi protein and beta-arrestin pathways. These receptors have been associated with chronic inflammation, asthma, and atherosclerosis [37,38].

### 3.4. Potential Targets of Natural Products Predicated by a Deep Learning Model, FusionDTA

As far as we know, this is the first attempt at large-scale molecular docking between all-natural products in the LOTUS database and targets applied to clinical drugs. Through the large-scale affinity prediction, we aimed to provide a relatively comprehensive list as a reference for the potential target space of natural products. Such data can provide references for the exploration of new functions of natural products. In brief, the result indicated that long-chain fatty acyls and tryptophan alkaloids had a wide range of targets. This large-scale prediction of affinity between natural products and therapeutic targets provided a new possibility for finding new functions of natural products. However, the reliability of active targets here still needs further verification.

## 4. Materials and Methods

### 4.1. Database

LOTUS natural product database (MongoDB dump) was downloaded from https://lotus.naturalproducts.net/download and accessed on 15 February 2023 [9]. The SQLite database of ChEMBL (release 32) was downloaded from https://ftp.ebi.ac.uk/pub/databases/ChEMBL/ChEMBLdb/latest/ on 15 February 2023 [8]. The sequence data for successful targets was retrieved from TTD via https://db.idrblab.net/ttd/full-data-download on 25 February 2023 [11].

### 4.2. Python Packages and Software

All data analyses were carried out with Python 3.11.1. The MongoDB dump was restored on the platform of MongoDB (version 4.4.6) and connected with pymongo (version 4.3.3) package. The source code of FusionDTA [10] was acquired from GitHub (https://github.com/yuanweining/FusionDTA) on 25 February 2023. The author-trained model of FusionDTA was retrieved from https://drive.google.com/file/d/1FfFLPhM2-97qvgkzcTiU30PluRPCm6vU/view?pli=1 on 25 February 2023. The chemical structures proceeded with rdkit release September 2022.

### 4.3. LOTUS Natural Product Data Preparation

After loading the MongoDB dump of LOTUS locally, we used pymongo to connect to the database and get the fields of lotus_id, chemicalTaxonomyNPclassifierPathway, chemicalTaxonomyNPclassifierSuperclass. The last three fields of chemical classification were renamed as pathway, superclass and class to facilitate subsequent calls and then stored in a file-based SQLite3 structure database named iNP.

To supplement the structure type information of the natural products, we used the urllib3 module to connect to the NPClassfier API interface via GET method (https://npclassifier.ucsd.edu/classify?smiles=<SMILES>) and upload smiles strings to supplement the structure type information, accessing on 17 February 2023. The special characters %, +, # and / in the SMILES string have special meanings in the URL, so they were replaced with %25, %2B, %23 and %2F before being assembled into the entire URL transfer message. Chemicals for which structural class information is unavailable were defined as “Other” types.

### 4.4. Extract and Summary of LOTUS Taxon Information

We used pymongo to connect to the LOTUS local database and get the lotus_id and taxonomyReferenceObjects fields. A Python script was then used to extract the kingdom, family, and genus information record-by-record and stored it in the SQLite3 database iNP. Then, we read the left join table of the kingdom and lotus NP table through pandas to obtain the corresponding data of pathway and kingdom field and present the number of natural products of various pathways occurring in each kingdom through a seaborn heatmap diagram.

### 4.5. Distribution Analysis of Natural Products Activity Data

ChEMBL is a freely accessible database that collects medicinal chemistry information and insights throughout the entire process of pharmaceutical research and development [8]. This database provides information on small molecules and their biological activity, sourced from full-text articles in medicinal chemistry journals, approved drugs, and clinical development candidates. It contains a vast collection, comprising over 15 million bioactivity measurements, 1.8 million annotated compounds with data on over 1600 cell lines, 500 tissues/organs, and 3600 organisms, all measured against 8200 proteins.

We obtained the activity screening data for natural products in ChEMBL by combining the data tables in the ChEMBL database named activities, assays, target_dictionary, target_components, component_class, protein_classification, and compounds_structures. These data tables were joined up by a SQL script and filtered out natural product entries associated with the lotusNP table extracted above. The result data table was sorted by pchembl_value in descending order. Duplicate values were removed based on the lotus_id and tid fields. After removing the redundant columns, the result data (354,795 entries) between the natural product and targets were stored in the np_activities table in the iNP database.

The pChEMBL of more than 8.0 was used as the threshold for classifying high activity. The lotusNP data table was then combined to obtain a data table containing the pathway, superclass, class of natural products, and target protein class information. Then, this data information was visualized on a network plot (igraph), replot (seaborn), and joyplot (joypy).

### 4.6. Predicting Affinities of Natural Products and Drug Targets

The source code of FusionDTA [10] was deployed on a workstation equipped with a display card of NVIDIA RTX A4000. FusionDTA runs in an environment with Python 3.7.3 associated with pytorch 1.13.1 and CUDA 11.7.1. FusionDTA does not accept conformational information for small molecules. Therefore, the SMILES strings for LOTUS natural products were de-configured using rdkit and deduplicated to yield 137,363 entries. The sequences of TTD successful targets (622 entries) were used as protein targets. All the target sequences were handled by esm-1b [39] to generate distributed contextual vectors as protein presentation for FusionDTA. The ligand–protein affinity was predicted for each drug–target combination with the best knowledge distillation model of Fusion DTA validated by the Davis dataset [40]. The predicted results were gathered and saved into a binary file.

## 5. Conclusions

Overall, we jointly analyzed the LOTUS natural product database, ChEMBL database, and TTD to sort out the intrinsic connections between natural product biogenic structure types, source species, and biotarget classes through data integration, cleaning, and systematic prediction by the deep learning model FusionDTA. Our analysis revealed that terpenoids, phenylpropanoids, and alkaloids are the most diverse family of natural products. These three major groups of natural products were mainly found in Archaeplastida, Plantae, and Viridiplantae. Polyketides were more commonly found in fungi, and peptides were common in bacteria. In the target space, alkaloids focused on neurological effects. Oligopeptides and polyketides were mainly associated with protein phosphorylation and HDAC receptors. Fatty acid readily intervenes in various physiological processes involving prostanoids and leukotrienes. We depicted the connections between species taxon, chemical structure groups, and biological targets through data analysis and visualization. Our data exploration provided a global perspective from understanding the connection profiled of natural products biogenic distribution, target space, and activity. We also look forward to more and better deep learning models to help us better understand the chembological relationship of natural products.

## Data Availability

The source code and data of this study are available at https://github.com/zq-lab/npTAnal.

## Supplementary Materials

The following supporting information can be downloaded at: https://www.mdpi.com/article/10.3390/ijms241411265/s1. We have organized the data in an Excel file to facilitate data reuse.
